# Editorial: Animal models and transgenic technology in Craniofacial biology

**DOI:** 10.3389/fphys.2023.1304715

**Published:** 2023-10-16

**Authors:** Junichi Iwata

**Affiliations:** ^1^ Department of Diagnostic and Biomedical Sciences, The University of Texas Health Science Center at Houston, School of Dentistry, Houston, TX, United States; ^2^ Center for Craniofacial Research, The University of Texas Health Science Center at Houston, School of Dentistry, Houston, TX, United States; ^3^ MD Anderson Cancer Center UTHealth Graduate School of Biomedical Sciences, Houston, TX, United States

**Keywords:** craniofacial development, genetics, animal models, dental diseases, environmental factors

Craniofacial deformities are one of the most common congenital birth defects (Iwata, 2021; Iwaya et al., 2023). Several important milestones in developing concepts, technologies, and methodologies in craniofacial biology have been achieved using animal models and transgenic technology. In fact, an increasing number of genetic and epigenetic studies show temporospatial mechanisms underlying craniofacial development by using animal models such as genetically-engineered animal models and chemical-induced disease models (Suzuki et al., 2016). In this Research Topic, we aimed to better understand the players and molecular mechanisms that are crucial for craniofacial/dental/oral development and diseases. Overall, a total of 5 articles (2 original and 3 review articles) was published in this Research Topic.

Alcohol consumption by pregnant mothers is known to increase the risk of various birth defects, including craniofacial anomalies (Iwata, 2021). Ghosal et al. showed that embryonic ethanol exposure causes a variety of craniofacial anomalies in *zebrafish* larvae. Notably, the development of each craniofacial tissue is independently affected by ethanol even though these tissues develop at the same stage and/or in a nearby space. Importantly, the authors found that craniofacial soft tissue development, including craniofacial neuromuscular integration, was particularly susceptible to ethanol-induced damages. Recent studies indicated that some environmental factors lead to epigenetic changes in craniofacial development, as reviewed in (Iwata, 2021). Mohd-Yunus et al. summarized the current knowledge on microRNAs, which are short single-stranded noncoding RNAs, in medication-related osteonecrosis of the jaw (MRONJ), a serious jaw bone injury related to certain medications used to treat osteoporosis or cancer. To date, there is no biomarker to aid in the diagnosis of this condition. In their review article, the authors discussed potential pathogenic microRNAs that diminish or augment osteoclast functions in MRONJ and bone resorption. Thus, epigenetic factors contribute to both development and disease through fine-tuned cell signaling pathways crucial for craniofacial development and functions (Suzuki et al., 2016). For example, both the disruption and augmentation of bone morphogenic protein (BMP) signaling are known to lead to craniofacial anomalies, as discussed in this Research Topic by Ueharu and Mishina. The authors reviewed the role of BMP signaling in craniofacial development and discussed the molecular mechanisms of fate determination and differentiation in cranial neural crest cells, which are multipotent cells that can give rise to various cell types and form craniofacial structures and tissues. In order to study such multipotent progenitor and stem cells, new tools and methodologies to help identify the mechanism(s) of fate determination and differentiation in these cells are necessary. Hsu and Maruyama introduced methodologies that are useful for studying skeletal stem cells, such as capsule transplantation and *ex vivo* culture systems, and shed light on the challenges and current limitations that accompany their application in stem cell research. In combination with transgenic animal models, these methodologies will bring many advantages to the field of craniofacial biology.

This Review Topic also introduces the use of animal models and transgenic approaches in the study of pathogenic mechanisms. For instance, Ohyama et al investigated the mechanism of dentinal pain using Wister rats and genetically-engineered mice Ohyama et al. Dentin hypersensitivity has been recognized as a significant oral health issue and shows a high prevalence of 8%–57% worldwide (Aminoshariae and Kulild, 2021). Ohyama et al showed that sensory transduction between odontoblasts (sensory receptor cells) and neurons is regulated through the Piezo1 (a mechanosensitive ion channel protein)—Pannexin 1 (a homo-heptameric membrane channel)—P2X_3_ receptor axis in dentinal sensitivity.

Overall, these original research and review articles published in this Research Topic will stimulate our continuous efforts for better understanding the molecular mechanisms underlying craniofacial developmental defects and diseases, which will lead to the development of diagnosis, treatment, and prevention approaches for these conditions.
